# Compassion Fatigue in a Cohort of South Italian Nurses and Hospital-Based Clinical Social Workers Following COVID-19: A Cross-Sectional Survey

**DOI:** 10.3390/jcm13144200

**Published:** 2024-07-18

**Authors:** Rosaria De Luca, Mirjam Bonanno, Maria Grazia Maggio, Antonino Todaro, Carmela Rifici, Carmela Mento, Maria Rosaria Anna Muscatello, Milva Veronica Castorina, Paolo Tonin, Angelo Quartarone, Maria Elena Pugliese, Rocco Salvatore Calabrò

**Affiliations:** 1 IRCCS Centro Neurolesi “Bonino-Pulejo”, Cda Casazza, SS 113, 98124 Messina, Italy; rosaria.deluca@irccsme.it (R.D.L.); mariagrazia.maggio@irccsme.it (M.G.M.); antonio.todaro@irccsme.it (A.T.); carmela.rifici@irccsme.it (C.R.); milva.castorina@irccsme.it (M.V.C.); angelo.quartarone@irccsme.it (A.Q.); roccos.calabro@irccsme.it (R.S.C.); 2 Psychiatry Unit, Policlinico Universitario “Gaetano Barresi”, 98124 Messina, Italy; cmento@unime.it (C.M.); mmuscatello@unime.it (M.R.A.M.); 3 Department of Biomedical and Dental Sciences and Morphofunctional Imaging, University of Messina, 98124 Messina, Italy; 4 Sant’Anna Institute, 88900 Crotone, Italy; patonin18@gmail.com (P.T.); me.pugliese@isakr.it (M.E.P.)

**Keywords:** long COVID, compassion fatigue, neurorehabilitation units

## Abstract

**Background/Objective**: The COVID-19 pandemic has led to a significant increase in the workloads of healthcare workers (HCWs). The fear of contracting the new virus with the frequent medical consequences has affected their mental health. As a result, they are at high risk of compassion fatigue (CF). In this multicentric study, as a primary objective, we evaluate the incidence and/or prevalence of CF in a cohort of Italian nurses and HCWs (hospital-based clinical social workers of neurological patients) who have contracted SARS-CoV-2 infection. Our secondary aim is to evaluate the difference in experiencing CF between subjects with and without long-term COVID. **Methods**: In this study, 101 HCWs attending three different neurorehabilitation settings (the Neurorehabilitation Unit of the “Bonino Pulejo” Neurolesi Center of Messina, the Neurorehabilitation Department of Crotone, and the Psychiatric Unit of the University Hospital of Messina) were enrolled from May 2021 to May 2023. Data were collected through self-administered semi-structured interviews. **Results**: We observed high percentages of CF difficulties in both nurses and HCWs, related to mood alteration in 57.7%, headaches in 44.4%, and fatigue in 62%. Higher percentages were found in individuals with long-term COVID-19, including mood alteration in 93.9%, headache in 88.6%, and memory-related problems in 98.5%. **Conclusions**: The complexity of a patient’s care pathway, especially in chronic disease situations, requires an enormous commitment that can lead to burnout and CF, which should be considered to initiate preventive interventions aimed at helping “those who help”, for the well-being of patients, healthcare teams, and healthcare organizations.

## 1. Introduction

Compassion fatigue (CF) is a complex concept that is characterized by the inability to cope with emotional stress, due to long-term exposure to caregiving and suffering people. In the past, CF has been identified in patients’ caregivers suffering from post-traumatic stress syndrome. In 1992, Carla Joinson [1] adapted, for the first time, the concept of CF to healthcare professions. In general, most of the theories about CF are based on Figley’s concept of CF, which is defined as “a state of exhaustion and dysfunction, biologically, physiologically, and emotionally, resulting from a prolonged exposure to compassion stress” [2,3]. During the care process, CF can be explained as a phenomenon that can occur when the amount of compassion exceeds the capacity to cope or recover [4]. Healthcare professionals, such as nurses, may suffer from CF and have difficulty adequately caring for their patients, as caring for seriously ill patients is often a trigger for CF. For these reasons, CF is experienced by nurses, doctors, psychologists, and other health professionals, who may experience reduced work performance, tiredness (emotional and physical), stress, and depression [5]. CF is very similar to burnout and post-traumatic stress disorder and can have a relevant impact on the psyche and daily life, especially in the workplace [6,7]. Notably, CF differs from burnout in that it results directly from observing the pain of others, whereas burnout can develop without this exposure. In any case, Hinderer et al. [5] observed that CF often shares the same risk factors as those for burnout, such as hours per shift, work relationships, and coping mechanisms. Indeed, CF was more likely to occur in nurses who reported fewer hobbies, weaker coworker relationships, 12 h rather than 8 h shifts, and the use of medicinal substances. Furthermore, they observed that 27.3% of nurses exposed to trauma reported CF, leading to various difficulties. Indeed, CF has a profound impact on the emotional and cognitive well-being of healthcare professionals. Psychologically, CF manifests itself through significant emotional exhaustion and cognitive difficulties [7,8]. Figley describes CF as a state in which caregivers experience symptoms such as intrusive thoughts and intense emotional distress, despite not having personally experienced the trauma [2,3]. The emotional symptoms of CF also include increased feelings of anxiety and depression, as “caregivers” may have difficulty managing their mental health while attending to the needs of others. Thus, the most common CF difficulties concern the emotional, physical, cognitive, social, and occupational levels [8,9,10].

In detail, the emotional feelings are anger, apathy, cynicism, discouragement, irritability, decreased enthusiasm, a perception of being overwhelmed, and intrusive flashbacks of experiences with and from patients. These are due to the extreme emotional load connected to the profession [11]. Cognitive difficulties, such as a reduction in attention and memory impairment, are also relevant, increasing the risks of medical errors. On the other hand, the physical symptoms are often fatigue and feelings of malaise [12]. Finally, the social difficulties are related to relationships with colleagues and patients, which can be heavily damaged by alienation, isolation, and indifference. Obviously, in a healthcare team, the presence of these difficulties can affect the achievement of healthcare goals and have a negative impact on the psychophysical well-being of the subject [13]. These situations can affect the “organizational” aspects of their work, due to absenteeism, decreased performance (mistakes in medications, errors in archiving and documentation), stereotyped communication, delays in schedules and deadlines, and finally, the avoidance of intense situations with patients [14]. Nurses and hospital-based clinical social workers (HCWs) are used to dealing with suffering and are often overloaded with work. Nonetheless, witnessing complex situations reported by patients, especially during the COVID-19 pandemic, could further negatively affect psychological well-being, with the possibility of experiencing CF [15,16].

Indeed, healthcare personnel may be affected by the consequences of the pandemic in two ways: (i) indirectly, linked to observing the suffering of patients isolated due to the virus; (ii) directly, due to the fear of contracting the virus [17]. Indeed, Haefner et al. [17] highlighted that healthcare professionals faced conflicting professional values, such as balancing their fear of contracting the virus and potentially transmitting it to family and friends, with the responsibility of providing effective care to patients and supporting their families. These moral and ethical dilemmas can cause mental health problems for some healthcare workers, leading to CF [17].

Moreover, the frequent permanence of symptoms beyond the acute phase of the infection, i.e., the presence of long-term COVID-19, should also be considered [18]. Symptoms of long-term COVID-19 can include muscle weakness, loss of smell and taste, fatigue, brain fog, shortness of breath, anxiety, and depression [19,20]. Hyassat et al., in a cohort study of 140 healthcare staff, observed that more than half of them (59.3%) reported long-term COVID-19 syndrome, with a prevalence among females compared to males (79.5% vs. 20.5%), and the most frequently reported symptom was fatigue [10]. However, the incidence and/or prevalence of CF in nurses and HCWs affected by long-term COVID is still unclear.

For these reasons, in this study, we primarily evaluate the presence of difficulties related to CF in a cohort of Italian nurses and HCWs who have suffered from SARS-CoV-2 infection.

Our secondary aim is to investigate the difference in experiencing CF between subjects with and without long-term COVID.

## 2. Materials and Methods

### 2.1. Study Design and Data Collection

**Figure 1:**
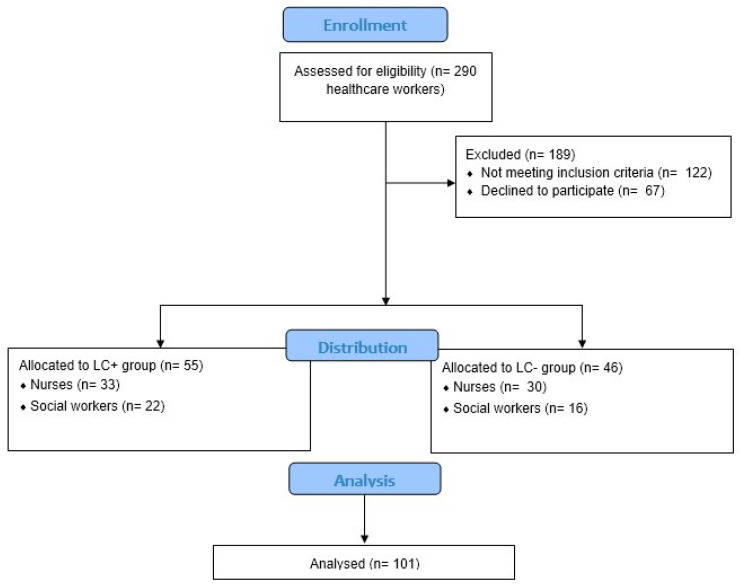
Flow chart for patient inclusion in cross-sectional studies, adapted from CONSORT diagram 2010.

The data were collected between May 2021 and May 2023, through a preliminary survey, and then, questionnaires were provided to nurses and HCWs of three different hospitals in southern Italy, including the Neurorehabilitation Unit of the “Bonino Pulejo” Neurolesi Center of Messina, the Sant’Anna Hospital of Crotone, and the Neuropsychiatry Service of the University Hospital of Messina (see Figure 1).

The eligibility criteria were as follows: (i) at least 1 year of work experience in neurorehabilitation settings (to validate patients with whom participants came into contact); (ii) no ongoing medical treatments for mental problems (e.g., antidepressants and anxiolytics) in the month before the administration of the questionnaire; (iii) previous SARS-CoV-2 infection, based on a positive rapid nasal swab or, in doubtful cases, a molecular swab that definitively proved the presence of the infection. We considered eligible participants who had contracted previous SARS-CoV-2 infection(s) at least 2 months before their enrolment. In addition, we decided to include participants with previous COVID-19 symptoms which lasted for 5 days. Otherwise, we included long COVID participants who manifested COVID-19 symptoms 3 months after negativization.

Among the participants who met these eligibility criteria, informed consent was requested, and the questionnaire was administered only to those who provided it. All participants gave their informed consent and socio-demographic data before completing the questionnaire. The informed consent form included a brief description of the purposes of the research and the questionnaire, the confidentiality of the information, the rights of the participants, and the contact information of the researchers. In particular, the explanatory document contained Figley’s definition of CF [2,3]. In addition, concrete examples were presented, showing the difficulties of CF in certain behaviors or emotions [2,3]. Participants filled out the questionnaires anonymously with a pencil and paper.

### 2.2. Procedures

**Figure 2:**
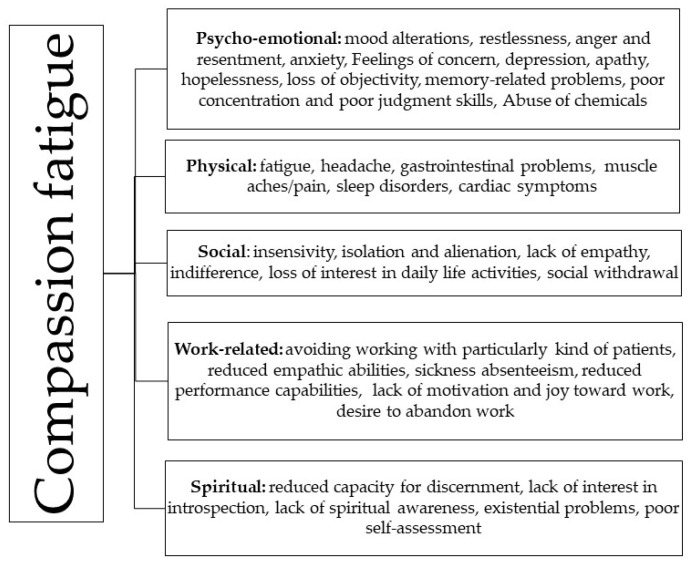
The list of CF-related symptoms reported on the questionnaires, for each category.

First, dedicated staff, including a psychiatrist, a psychiatric rehabilitation technician, and a psychologist, administered preliminary interviews to nurses and HCWs. Second, they provided them with a questionnaire comprising two parts: (1) socio-demographic data, including the nurse’s or HCW’s position, age, gender (male or female), education level according to the Italian educational system (middle school, high school, university, master’s degree), number of times they had contracted SARS-CoV-2 infection (e.g., once, twice, or three or more times); (2) CF subdomains [21,22], which included a five-category checklist related to CF difficulties (i) psycho-emotional, (ii) physical, (iii) social, (iv) work-related, and (v) spiritual. According to Figley’s definition of CF [2,3], each category comprises a range of different difficulties that need to be marked with a sign, if the individual has the difficulties reported. In addition, we considered it to be a dichotomic scale, using “Yes” (presence) and “No” (absence). Consequently, we transformed the answers into proportions and percentages (see Figure 2).

After the preliminary interview, the investigators asked each participant to fill in both parts of questionnaire and to tick off the prevalent difficulties for each domain investigated [2,3,10,23].

In addition, the diagnosis of Long COVID syndrome, defined as the persistence of COVID-19 symptoms for at least two months following infection with SARS-CoV-2 [24], was carried out through an in-depth anamnesis, and the specific questionnaire used to diagnose long COVID was the “Post-COVID-19 Functional Status” questionnaire, consisting of an ordinal scale ranking patients’ answers in meaningful categories [25,26].

### 2.3. Ethical Considerations

The data collected in this study was conducted in accordance with the ethical standards of the institutional and/or local ethical research committee (IRCCS-ME-69/2021) and with the Declaration of Helsinki. Informed and written consent was obtained from all participants prior to their inclusion in the study.

### 2.4. Data Analysis

A descriptive analysis of the sample was conducted, determining the age, gender, educational level, occupation, and instances of SARS-CoV-2 infection for both nurses and HCWs with and without LC (see Table 1 and Table 2). Continuous variables were expressed as mean and standard deviation, while categorical data, including educational level, profession, history of SARS-CoV-2 infection, and questionnaire responses, were articulated in terms of frequencies and percentages.

The normality of the variables was assessed using the Kolmogorov–Smirnov test. Given the non-normal distribution of the data, non-parametric analysis was employed. The Mann–Whitney U test was utilized for comparing two independent groups, specifically, when the dependent variable was either ordinal or continuous but not normally distributed. Subsequently, the χ^2^ test with continuity correction was applied to evaluate statistical disparities in proportions across categorical variables.

This continuity correction involves adjusting the test statistic to account for the discrete nature of the data and the approximation of the chi-squared distribution. The continuity correction is implemented by subtracting 0.5 from the absolute difference between each observed and expected cell frequency before squaring and summing these differences in the chi-squared statistic [27]. This correction helps improve the approximation of the test statistic to a chi-squared distribution, especially when dealing with small sample sizes or sparse data. The whole analysis was performed in the open-source software R 4.1.3. (Vienna, Austria) [28]. For all hypotheses tested, two-tailed *p*-values less than 0.05 were considered significant.

## 3. Results

A sample of 101 nurses and HCWs returned their filled-in questionnaires (see Supplementary Tables S1 and S2). Of 101 subjects, 46 individuals (33 females and 13 males), with a mean age of 43.78 ± 11.03, had a history of SARS-CoV-2 infection but no symptoms of long COVID (LC−), whereas 55 individuals with a mean age of 44.91 ± 10.9 (42 females and 13 males) showed symptoms of long COVID (LC+). Specifically, LC− participants were made up of 30 (65.21%) nurses and 16 (34.78%) HCWs, while participants with LC+ included 33 (%) nurses and 22 (%) HCWs. In addition, we found that the distribution regarding educational level was different between nurses and HCWs (*p* < 0.03). In fact, regarding HCWs, only 7 out of 38 graduated from university; otherwise, nurses had mostly graduated and also had a master’s degree. For more details see Table 1 and Table 2.

### 3.1. Primary Aim

As reported in Table 3, in nurses reporting LC+, there were high rates of psycho-emotional and cognitive difficulties, such as alterations in mood (87.9%), restlessness (81.8%), irritability (80.6%), anger and resentment (48.5%), anxiety (51.5%), feelings of worry (51.5%), depression (45.5%), memory problems (97%), and poor concentration and judgment skills (75.8%). We also found physical difficulties, like fatigue (66.7%), headaches (81.8%), muscle pain (54.5%), sleep disturbances (69.7%), and cardiac symptoms (42.4%). In addition, nurses also complained of social difficulties related to CF, such as isolation and alienation (30.3%) in association with a loss of interest in activities of daily living (24.2%) and social withdrawal (25%). Finally, working-related difficulties among nurses included a lack of motivation and joy toward work (36.3%), as well as a desire to abandon work (36.3%).

In HCWs, especially those with LC+, we noticed high levels of “yes” responses to psycho-emotional and cognitive difficulties: alterations in mood (100%), restlessness (90.9%), irritability (77.3%), anger and resentment (50%), anxiety (86.4%), feelings of worry (43.8%), memory problems (100%), and poor concentration and judgment skills (100%). In addition, they also showed physical difficulties, like headaches (95.5%), muscle pain (59.1%), and sleep disturbances (87.5%), although they did not perceive high levels of chronic fatigue (50% for LC+ and 0% for LC−), unlike nurses. Furthermore, we found that the social, work-related, and spiritual difficulties of CF in HCWs (both LC+ and LC−) received fewer affirmative responses than in nurses (see Table 3).

### 3.2. Secondary Aim

Generally, we found that both nurses and HCWs with LC+ showed higher rates of “yes” responses in nearly all the different CF difficulty categories (e.g., psycho-emotional, physical, social, work-related, and spiritual). In detail, in nurses, we observed statistically significant differences between the LC− group and LC+ group in the presence of CF difficulties, especially for the psycho-emotional and cognitive difficulties related to CF, such as memory-related problems (*p* < 0.001) and poor concentration and judgment skills (*p* < 0.001) (see Table 3). Indeed, we found significant statistical differences in physical difficulties between LC- and LC+ nurses regarding headaches (*p* < 0.002), sleep disturbances (*p* < 0.001), and cardiac symptoms (*p* < 0.001). It is noteworthy that physical difficulties in LC− nurses were not a concern, except for fatigue (61.9%) and headache (45.5%). Among the social difficulties related to CF, isolation and alienation (*p* < 0.004) was the one that showed a statistically significant difference between LC- and LC+ nurses.

Similarly, among HCWs, nurses showed statistically significant differences between LC− and LC+ in psycho-emotional items, like mood alteration (*p* < 0.004), restlessness (*p* < 0.001), irritability (*p* < 0.001), and cognitive difficulties related to CF, such as memory-related problems (*p* < 0.001) and poor concentration and judgment skills (*p* < 0.001). Additionally, chronic fatigue (*p* < 0.001), headache (*p* < 0.001), and sleep disorders (*p* < 0.001) resulted the most significant differences between LC− and LC+ in HCWs. Regarding social symptoms in HCWs, we found that a loss of interest in the activities of daily living (*p* < 0.003) was the one that showed statistical significance between LC− and LC+ (see Table 3).

## 4. Discussion

To the best of our knowledge, this is the first study that has investigated the presence of CF in a sample of nurses and HCWs with and without LC.

We observed high incidence rates of CF difficulties in most nurses and HCWs, especially in individuals with long-term COVID. The most reported difficulties by our sample were psycho-emotional (i.e., mood alteration, restlessness, irritability, anxiety), cognitive (i.e., memory-related problems and poor concentration and judgment skills), and physical (i.e., headaches, and muscle pain) ones. CF affects cognitive and psychological functioning, leading to difficulties in clear thinking, judgment, decision-making, and concentration. It can cause memory lapses and contribute to a negative self-image and feelings of inadequacy and helplessness. These changes have significant consequences, contributing to a range of stress-related physical and psychiatric disorders. In the short term, CF can cause physical health issues related to high cortisol levels, which can increase susceptibility to illness [29]. Additionally, we noticed that nurses perceived more social and work-related difficulties than HCWs. This is probably related to their role in the organization in addition to poor knowledge about CF and reduced coping strategies [30]. Indeed, HCWs are less exposed to organization and management concerns, and this could explain the fewer “yes” responses among HCWs for the social and work-related CF dimensions. CF can induce an inclination to avoid patients and situations. HCWs and nurses who experience CF may no longer find enjoyment in work, leading to a decrease in efficiency and self-esteem. Declines in efficiency, productivity, and professional competence, along with the increased risk of medical errors, can lead to dismissal and even career termination. These aspects have been further worsened by COVID-19 infection and all of its consequences. The COVID-19 pandemic has negatively impacted the psychological health and well-being of healthcare providers. An amplification in chronic stressors, workload, and fatalities may have increased the risk of CF and disrupted the quality of patient care. Several articles have indeed confirmed that COVID-19 had a major impact on nurses and HCWs [31,32,33], with significant psychological discomfort, especially in nurses, as observed in our sample. Furthermore, nurses are more exposed to different stressors, such as reassignment to COVID-19 units, fear of contaminating their loved ones, and higher turnover, than HCWs [34]. Indeed, various studies have identified significant levels of anxiety and depression for nurses following COVID-19 in their work environments, with a negative impact on their daily personal and professional lives [34,35]. Hickling et al. found that nurses may experience moderate to severe levels of anxiety, mild or severe depression, and difficulties consistent with diagnoses of post-traumatic stress disorder, characterized by recurring intrusive recalls and recollections of work-related stressful events [36]. These experiences could be encouraged by the coexistence of one’s symptoms due to long-term COVID, which reduces the mental, emotional, and physical abilities of nurses and HCWs.

A recent survey of long-term COVID in 603 physicians found that participants reported a wide range of long-term COVID symptoms, such as fatigue, headaches, muscle aches, nerve damage, and aches and pains, as well as constant breathing problems. These issues have significant implications for patient care and healthcare performance, as the authors noticed that 18% of participants with long-term COVID said they were unable to work, and only 31% of healthcare professionals declared that they worked full-time [37]. In this sense, the workload, the long-term COVID symptoms experienced, the fear of contagion, and the job demands linked to this crisis, with constant exposure to death and the fear of being infected, leads to continued exposure to traumatic stimuli, with a greater probability of experiencing CF [32]. According to Joinson, CF is a particular form of burnout that affects caregivers [1]. It happens when operators assist patients, identifying with their trauma and pain, so they use all their energy to help patients, neglecting their own needs [38]. As a result, nurses and HCWs may experience physical, emotional, social, and spiritual exhaustion, resulting in a decline in compassion and empathy, and reduced energy [39]. Moreover, nurses and HCWs can experience burnout, which, according to Maslach, is a syndrome involving tiredness, a sense of impotence, despair, and negative attitudes toward one’s work and patients [40]. Chesi et al. conducted a study to evaluate the quality of life of one hundred and five neurologists and nurses from 30 Italian centers employed in a multiple sclerosis ward. The authors observed that the nurses and HCWs suffered from CF and work discomfort due to the patients’ continued exposure to suffering and distress. These factors can cause a low sense of accomplishment and severe emotional exhaustion [41]. We are not able to completely explain the reason why nurses and HCWs with LC+ experienced more CF than those without. Some may be concerned that there is an overlap between long COVID and CF, since it is not easy to differentiate, e.g., fatigue due to LC from that related to CF. Therefore, specific investigations are needed to help clinicians better diagnose these two overlapping syndromes.

## 5. Clinical Implications

This study highlights some relevant clinical implications for the management of CF among nurses and other healthcare workers, particularly for those who have suffered from long-term COVID-19. Our findings reveal that healthcare professionals with persistent COVID-19 symptoms (LC+) experience significantly higher rates of psychological, emotional, cognitive, and physical difficulties related to CF compared to their colleagues without long-term COVID-19 (LC−). This suggests that long-term COVID-19 may intensify CF symptoms, leading to increased issues such as mood changes, difficulty concentrating, and headaches among healthcare professionals. Consequently, it is crucial to implement targeted support strategies and stress management interventions to mitigate the impact of CF and enhance the well-being of healthcare workers. As such, it has become essential to raise awareness and train staff on how to recognize and manage CF, especially in environments with high trauma like chronic neurological disorders, and long-term exposure.

Recognizing the signs of CF early is crucial for maintaining well-being and the ability to provide effective care. To achieve this, healthcare organizations could screen HCWs and nurses using questionnaires and then conduct interviews with specialized psychologists for those identified as being at greater risk, such as individuals with long-term COVID-19, for developing CF. This approach would enable the implementation of effective prevention measures for helping those who help. Practical interventions could include self-care, seeking support, maintaining a healthy lifestyle, practicing mindfulness and stress reduction, and prioritizing worker well-being through comprehensive care initiatives. Other significant interventions could include regular psychological support, wellness programs, and personalized coping strategies, thus helping to improve both the quality of patient care and the mental health of healthcare workers and nurses. Each of these aspects significantly impacts the well-being of neurological healthcare teams and healthcare organizations, ultimately enhancing the efficiency of care for patients with neurological disorders.

## 6. Limitation and Future Research

This study has some limitations that need to be acknowledged. First, the two samples analyzed were not large enough to be representative of the entire nurse and HCW population. Future larger studies with randomized samples could be beneficial to further investigate the extent of CF in healthcare workers in Italy. Second, there was a lack of follow-up, and there were no comparisons with pre-COVID-19 conditions. Third, we did not focus on the possible types of bias in the administration of questionnaires (such as recall bias, selection bias, etc.) that can be used as a checklist for identifying potential problem/errors (questions with problematic wording; questionnaires that are too long; the administration of questionnaires to populations with cultural differences) when designing and administering the questionnaire [42]. Finally, we did not consider job resource variables, such as support from colleagues and supervisors. In the future, new studies with a larger sample and follow-up would be useful to support the results obtained and provide adequate interventions to prevent situations that put nurses and HCWs at risk, considering the role of long-term COVID-19. Indeed, future research could focus on interventions to manage risk factors for CF among nurses and HCWs.

## 7. Conclusions

According to our study, CF is a very common symptom affecting nurses and HCWs and, therefore, a potential public health problem in different clinical settings, especially during pandemics. Nurses and HCWs suffering from Long COVID seem to be more affected by CF, and this issue deserves further investigation. The complexity of a patient’s care pathway, mainly in chronic neurological conditions, requires an enormous commitment that can lead to burnout and CF, which should be considered to initiate preventive interventions aimed at helping those who help, for the well-being of patients, healthcare teams, and healthcare organizations.

## Figures and Tables

**Table 1:** Demographic and clinical features of the nurse sample, stratified according to the presence of long-term COVID (LC) syndrome.

		All Nurses (N = 63)	LC− (N = 30)	LC+ (N = 33)	*p*-Value *
**Nurses**	**Age**	43.380 ± 10.648	41.60 ± 10.45	45.69 ± 11.01	0.245
	**Gender**				0.693
	*Females*	45 (71.4%)	22 (73.3%)	23 (69.7%)	
	*Males*	18 (28.5%)	8 (26.6%)	10 (30.3%)	
	**Educational level**				0.494
	*Middle school*	0 (0.0%)	0 (0.0%)	0 (0.0%)	
	*High school*	9 (14.2%)	5 (16.6%)	4 (12.1%)	
	*University*	39 (61.9%)	18 (60.0%)	21 (63.6%)	
	*Master’s degree*	15 (23.8%)	7 (23.3%)	8 (24.2%)	
	**Instances of SARS-CoV-2 infection**				0.061
	*Once*	57 (90.4%)	30 (100.0%)	27 (81.8%)	
	*Twice*	6 (9.5%)	0 (0.0%)	6 (18.2%)	

Legend: LC− (nurses without long COVID); LC+ (nurses with long COVID). * Quantitative variables are expressed as means ± standard deviations and were compared using the Mann–Whitney U test, whereas categorical variables are presented as frequencies and percentages and were compared using the χ^2^ test.

**Table 2:** Demographic and clinical features of the HCW sample, stratified according to the presence of long-term COVID (LC) syndrome.

	All Social Workers (N = 38)	LC− (N = 16)	LC+ (N = 22)	*p*-Value *
**Age**	44.34 ± 14.73	47.80 ± 11.29	46.18 ± 12.21	0.191
**Gender**				0.672
*Females*	30 (78.9%)	11 (68.7%)	19 (86.3%)	
*Males*	8 (21.0%)	5 (31.2%)	3 (13.6%)	
**Educational level**				0.302
*Middle school*	9 (23.6%)	3 (18.7%)	6 (27.2%)	
*High school*	20 (52.6%)	8 (50.0%)	12 (54.5%)	
*University*	7 (18.4%)	4 (25.0%)	3 (13.6%)	
*Master’s degree*	2 (5.2%)	1 (6.2%)	1 (4.5%)	
**Times of SARS-CoV-2 infection**				0.283
*Once*	30 (78.9%)	14 (87.5%)	16 (72.7%)	
*Twice*	8 (21.0%)	2 (12.5%)	6 (27.2%)	

Legend: LC− (social workers without long COVID); LC+ (social workers with long COVID). * Quantitative variables are expressed as means ± standard deviations and were compared using the Mann–Whitney U test, whereas categorical variables are presented as frequencies and percentages and were compared using the χ^2^ test.

**Table 3:** Statistical comparison, regarding the difficulties of CF, between both nurses and HCWs and the whole cohort with and without long-term COVID.

		Nurses *		*p*-Value **	Social Workers *		*p*-Value **	Whole Cohort *		*p*-Value **
Psycho-Emotional Symptoms of Compassion Fatigue		LC− (30) N (%)	LC+ (33) N (%)		LC− (16) N (%)	LC+ (22) N (%)		LC− (46) N(%)	LC+(55) N (%)	
**Psycho-emotional symptoms of compassion fatigue**	**Mood alteration**	14 (46.7)	29 (87.9)	<0.001 ***	11 (68.8)	22 (100.0)	0.004 ***	25 (54.3)	51 (92.7)	<0.001 ***
	**Restlessness**	8 (26.7)	27 (81.8)	<0.001 ***	6 (37.5)	20 (90.9)	<0.001 ***	14 (30.4)	47 (85.4)	**<0.001** ***
	**Irritability**	8 (26.7)	25 (80.6)	<0.001 ***	4 (25.0)	17 (77.3)	0.001 ***	12 (26.0)	42 (76.4)	<0.001 ***
	**Anger and resentment**	7 (23.3)	16 (48.5)	<0.001 ***	2 (12.5)	11 (50)	0.161	9 (19.5)	27 (49.0)	<0.001 ***
	**Anxiety**	6 (20)	17 (51.5)	<0.001 ***	6 (37.5)	19 (86.4)	0.001 ***	12 (26.0)	36 (65.4)	**<0.001** ***
	**Feelings of concern**	6 (20)	17 (51.5)	<0.001 ***	7 (43.8)	10 (43.8)	0.912	13 (28.2)	27 (49.0)	<0.001 ***
	**Depression**	0	15 (45.5)	<0.001 ***	0	4 (18.2)	0.713	0	19 (34.5)	**<0.001**
	**Apathy**	1 (3.3)	6 (18.2)	<0.001 ***	2 (12.5)	4 (18.2)	0.631	3 (6.5)	10 (18.1)	0.080
	**Hopelessness**	0	4 (12.1)	0.040	0	1 (4.5)	0.362	0	5 (9.0)	0.040
	**Loss of objectivity**	2 (6.7)	8 (24.2)	<0.001 ***	0	5 (22.7)	0.041	2 (4.3)	13 (23.6)	0.010
	**Memory-related problems**	6 (20)	32 (97.0)	<0.001 ***	2 (12.5)	22 (100)	<0.001 ***	8 (17.4)	54 (98.1)	<0.001 ***
	**Poor concentration and poor judgment skills**	3 (10)	25 (75.8)	<0.001 ***	1 (6.2)	22 (100)	<0.001 ***	4 (8.7)	47 (85.4)	<0.001 ***
	**Abuse of chemicals**	0	5 (15.2)	**<0.001**	0	1 (4.5)	0.382	0	6 (10.9)	0.020
**Physical symptoms of compassion fatigue**	**Fatigue**	13 (61.9)	22 (66.7)	0.720	0	8 (50.0)	<0.001 ***	13 (28.2)	30 (54.5)	<0.001 ***
	**Headache**	15 (45.5)	27 (81.8)	0.002 ***	7 (43.8)	21 (95.5)	<0.001 ***	22 (47.8)	48 (87.2)	<0.001 ***
	**Gastrointestinal problems**	1 (3.3)	9 (25.0)	0.014	2 (12.5)	7 (31.8)	0.163	3 (6.5)	16 (29.0)	<0.001 ***
	**Muscle aches/pain**	7 (23.3)	18 (54.5)	0.011	3 (18.8)	13 (59.1)	0.0124	10 (21.7)	31 (56.3)	<0.001 ***
	**Sleep disorders**	6 (20)	23 (69.7)	<0.001 ***	4 (25.0)	14 (87.5)	<0.001 ***	10 (21.7)	37 (67.2)	<0.001 ***
	**Cardiac symptoms**	0	14 (42.4)	<0.001 ***	1 (6.2)	6 (27.3)	0.98 1	1 (2.2)	22 (40.0)	<0.001 ***
**Social symptoms of compassion fatigue**	**Insensivity**	1 (3.3)	3 (9.1)	0.360	0	2 (9.1)	0.213	1 (2.2)	5 (9.0)	0.150
	**Isolation and alienation**	1 (3.3)	10 (30.3)	0.004 ***	1 (6.2)	7 (31.8)	0.055	2 (4.3)	17 (30.9)	<0.001 ***
	**Lack of empathy**	0	3 (9.1)	0.090	0	3 (13.6)	0.12 1	0	6 (10.9)	0.020
	**Indifference**	2 (6.7)	6 (18.2)	0.170	0	2 (9.1)	0.211	2 (4.3)	8 (14.5)	0.090
	**Loss of interest in daily life activities**	3 (10)	8 (24.2)	0.130	0	9 (40.9)	0.003 ***	3 (6.5)	17 (30.9)	<0.001 ***
	**Social withdrawal**	5 (16.6)	9 (25.0)	0.310	2 (12.5)	9 (40.9)	0.056	7 (15.2)	18 (32.7)	0.040
**Work-related symptoms of compassion fatigue**	**Avoiding working with particularly kind of patients**	0	6 (18.2)	**0.01**	1 (6.2)	2 (9.1)	0.742	1 (2.2)	8 (14.5)	0.030
	**Reduced empathic abilities**	2 (6.7)	2 (6.0)	0.920	0	4 (25.0)	0.071	2 (4.3)	6 (10.9)	0.230
	**Sickness absenteeism**	0	2 (6.0)	0.170	0	3 (13.6)	0.782	0	5 (9.0)	0.040
	**Reduced performance capabilities**	0	5 (15.2)	**0.02**	0	2 (9.1)	0.21 2	0	7 (12.7)	0.010
	**Lack of motivation and joy toward work**	0	12 (36.3)	**<0.001**	0	4 (25)	0.0713	0	16 (29.0)	<0.001 ***
	**Desire to abandon work**	2 (6.7)	12 (36.3)	**0.004**	0	7 (31.8)	0.0124	2 (4.3)	19 (34.5)	<0.001 ***
**Spiritual symptoms of compassion fatigue**	**Reduced capacity for discernment**	0	6 (18.2)	0.0100	1 (6.2)	6 (27.3)	0.098	1 (2.2)	12 (21.8)	<0.001 ***
	**Lack of interest in introspection**	0	4 (12.1)	0.040	0	6 (27.3)	0.022	0	10 (18.1)	<0.001 ***
	**Lack of spiritual awareness**	0	1 (3.03)	0.320	0	5 (22.7)	0.044	0	6 (10.9)	0.020
	**Existential problems and poor self-assessment**	1 (3.3)	5 (15.2)	0.110	1 (6.2)	6 (27.3)	0.98 2	2 (4.3)	11 (20.0)	0.020

Legend: LC− (without long COVID); LC+ (with long COVID). * The percentages represent the affirmative responses to the administered CF symptoms questionnaire, which were compared using a χ^2^ test between LC− and LC+. ** Significant *p*-values are in bold. *** *p*-level < 0.005.

## Data Availability

Data will be available on request to the corresponding author.

## Supplementary Materials

The following supporting information can be downloaded at: https://www.mdpi.com/article/10.3390/jcm13144200/s1; Table S1: Demographic and clinical characteristics of whole HCW cohort. Table S2: Demographic and clinical characteristics of all participants divided into LC− and LC+.
