# Supplementary material for: Compassion Fatigue in a Cohort of South Italian Nurses and Hospital-Based Clinical Social Workers Following COVID-19: A Cross-Sectional Survey
Source: J Clin Med. 2024 Jul 18;13(14):4200. doi: 10.3390/jcm13144200 (PMC11278230; doi:10.3390/jcm13144200)
Supplement: Supplementary file 1 [file jcm-13-04200-s001.zip › jcm-3067116-supplementary.pdf]

Supplementary Materials

Supplementary Table S1. Demographic and clinical characteristics of HCW whole cohort

Demographic Variables*	HCW whole cohort	Nurses	Social workers	p-value*
Sample size	101 (100.0%)	63 (62.4%)	38 (37.6%)	
Males	26 (25,7%)	18 (28.5%)	8 (21.0%)	
Females	75 (74.3%)	45 (71.4%)	30 (78.9%)	0.127
Age (years)				
**Educational level**	44.69 ± 11.12	43.38 ± 10.64	44.34 ± 14.73	0.508
Middle school	9 (8.9%)	0 (0.0%)	9 (23.6%)	
High school	29 (28.7%)	9 (14.2%)	20 (52.6%)	0.031
University	46 (45.5%)	39 (61.9%)	7 (18.4%)	
Master's degree	17 (16.8%)	15 (23.8%)	2 (5.2%)	
**Times of *SarS-COv-2 infection***				
Ones	87 (86.1%)	57 (90.4%)	30 (78.9%)	
Twice	14 (13.8%)	6 (9.5%)	8 (21.0%)	0.717

*The continuous variables expressed as mean ± SD and categorical variables as frequency (%).

** Quantitative variables were expressed as means ± standard deviations and were compared using the Mann–Whitney U test, whereas categorical variables were presented as frequencies and percentages and were compared using the χ^2 test.

Supplementary Table S2. Demographic and clinical characteristics of all participants divided into LC− and LC+.

Demographic Variables	LC –	LC +	HCWs Whole cohort	p-value
Sample size	46 (45.5%)	55 (54.5%)	101 (100.0%)	
**Nurses**	30 (65.2%)	42 (76.3%)	72 (71.3%)	0.46
**Social workers**	16 (34.8%)	13 (23.6%)	29 (28.7%)	
**HCWs whole cohort**	46 (45.5%)	55 (54.5%)	101 (100.0%)	
Males	13 (28.2%)	13 (23.6%)	26 (26.7%)	0.753
Females	33 (71.7%)	42 (76.3%)	75(74.3%)	
Age (years)	43.7 ± 11.0	45.45 ± 11.25	44.69 ± 11.13	0.454
**Educational l evel**				0.502
Middle school	3 (6.5%)	6 (10.0%)	9 (8.9%)	
High school	13 (28.3%)	16 (29.0%)	29 (29.8%)	
University	22 (47.8%)	24 (43.6%)	46 (45.5%)	
Master's degree	8 (17.4%)	8 (14.5%)	16 (15.8%)	
**Times of *SarS-COv-2 infection***				0.726
Ones	44 (95.6%)	43 (58.2%)	87 (86.1%)	
Twice	2 (4.4%)	12 (21.8%)	14 (13.9%)	

*The continuous variables expressed as mean ± SD and categorical variables as frequency (%).

** Quantitative variables were expressed as means ± standard deviations and were compared using the Mann–Whitney U test, whereas categorical variables were presented as frequencies and percentages and were compared using the χ^2 test.
